# Supplementary material for: Genetic evolution of keratinocytes to cutaneous squamous cell carcinoma
Source: Nat Commun. 2025 Nov 27;16:10663. doi: 10.1038/s41467-025-65687-y (PMC12660313; doi:10.1038/s41467-025-65687-y)
Supplement: Supplementary file 1 — Supplementary Information [file 41467_2025_65687_MOESM1_ESM.pdf]

Supplementary Information

Genetic evolution of keratinocytes to cutaneous squamous cell carcinoma

Authors: Bishal Tandukar, Delahny Deivendran, Limin Chen, Aravind K. Bandari, Noel Cruz-Pacheco, Harsh Sharma, Meng Wang, Albert Xu, Daniel B. Chen, Christopher D. George, Annika L. Marty, Raymond J. Cho, Jeffrey Cheng, Drew Saylor, Pedram Gerami, Iwei Yeh, Sarah T. Arron, Boris C. Bastian, A. Hunter Shain

This document contains **Supplementary Figures 1-14**

Figure S1

a

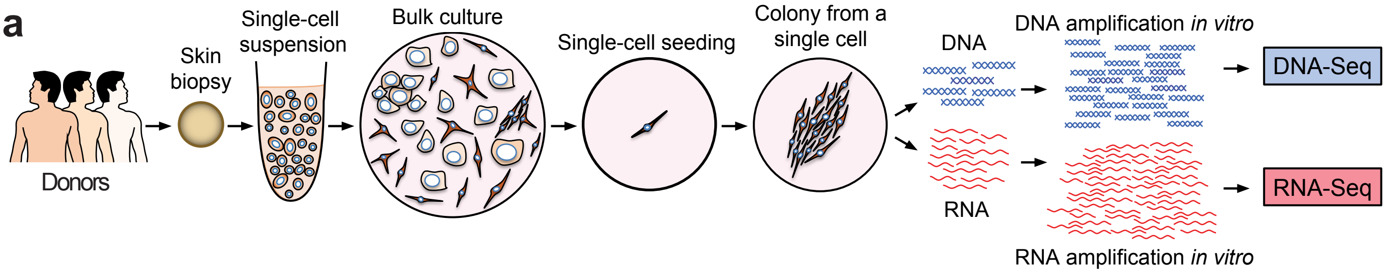

b

**Donor 6, Male, 64 yrs**<br>*L. Upper Back, R. Back*<br>- 3 keratinocytes<br>- 29 melanocytes<br>- 2 fibroblasts	**Donor 21, Male, 57 yrs**<br>*L. Ear*<br>- 4 keratinocytes<br>- 5 melanocytes<br>- 0 fibroblasts	**Donor 49, Female, 52 yrs**<br>*R. Lower Back*<br>- 8 keratinocytes<br>- 9 melanocytes<br>- 2 fibroblasts	**Donor 54, Male, 42 yrs**<br>*Shoulder, Buttocks*<br>- 20 keratinocytes<br>- 9 melanocytes<br>- 3 fibroblasts	**Donor 57, Female, 70 yrs**<br>*Face*<br>- 5 keratinocytes<br>- 10 melanocytes<br>- 3 fibroblasts
**Donor 13, Male, 74 yrs**<br>*Scalp, R. Earlobe, Upper Back*<br>- 4 keratinocytes<br>- 30 melanocytes<br>- 0 fibroblasts	**Donor 37, Female, 43 yrs**<br>*R. Lower Back*<br>- 4 keratinocytes<br>- 2 melanocytes<br>- 0 fibroblasts	**Donor 51, Female, 95 yrs**<br>*Scalp*<br>- 2 keratinocytes<br>- 0 melanocytes<br>- 0 fibroblasts	**Donor 55, Male, 70 yrs**<br>*Shoulder, Buttocks*<br>- 14 keratinocytes<br>- 7 melanocytes<br>- 3 fibroblasts	**Donor 58, Female, 75 yrs**<br>*Shoulder*<br>- 10 keratinocytes<br>- 3 melanocytes<br>- 2 fibroblasts
**Donor 14, Female, 35 yrs**<br>*Abdomen*<br>- 2 keratinocytes<br>- 2 melanocytes<br>- 0 fibroblasts	**Donor 38, Female, 40 yrs**<br>*R. Lower Back*<br>- 4 keratinocytes<br>- 6 melanocytes<br>- 1 fibroblast	**Donor 53, Female, 50 yrs**<br>*Shoulder, Buttocks*<br>- 19 keratinocytes<br>- 7 melanocytes<br>- 3 fibroblasts	**Donor 56, Male, 74 yrs**<br>*Face, Shoulder*<br>- 28 keratinocytes<br>- 3 melanocytes<br>- 3 fibroblasts	**Donor 59, Female, 56 yrs**<br>*Shoulder*<br>- 10 keratinocytes<br>- 9 melanocytes<br>- 1 fibroblasts

c

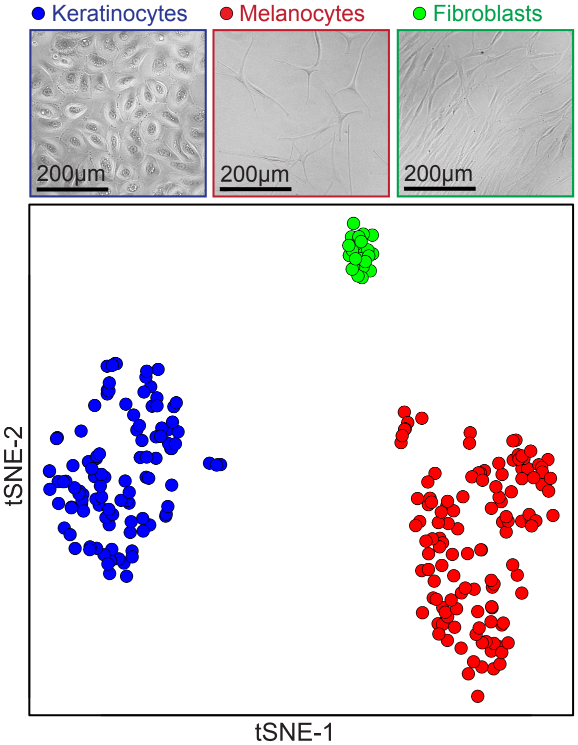

d

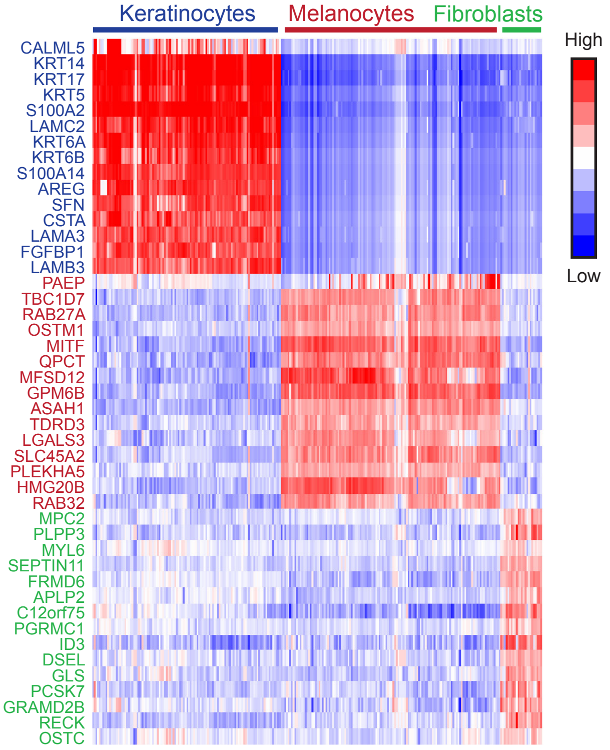

Figure S1. An approach to measure the mutational landscapes of individual skin cells, and a summary of cells genotyped in this study. a An overview of our single-cell genotyping workflow. **b** Summary of donors, biopsies, and cell types included in this study. **c** The lineage of each cell was confirmed from gene expression data of each colony. A t-SNE plot where each data point corresponds to a cell. Cells with similar gene expression profiles clustered together and are colored based on their morphological features (appearing as either keratinocytes, melanocytes, or fibroblasts) with a representative image from each group shown above the plot. **d** Differential gene expression analysis was performed, comparing the three groups of cells in panel c. The top 15 upregulated genes in each group are shown in the heatmap. Each column corresponds to a cell and each row to a gene, as indicated, with red/blue tiles depicting genes that are relatively up/down-regulated. The top genes in each group are consistent with genes known to be expressed in either keratinocytes, melanocytes, or fibroblasts. Source data are provided as a Source Data file.

Figure S2.

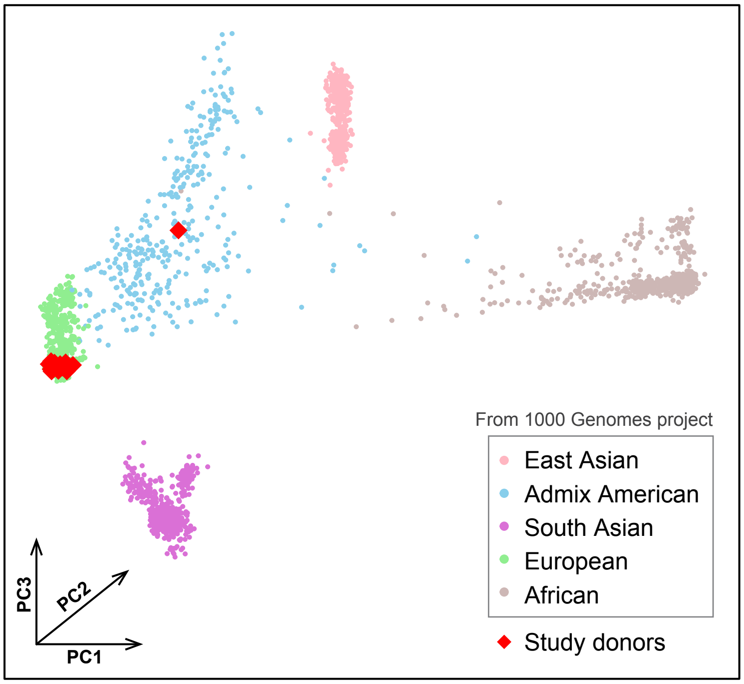

Figure S2. Genetic ancestry of the study donors. The principal component analysis (PCA) plot illustrates the genetic ancestry of the participating donors, with 14 donors clustering with individuals of European ancestry and 1 donor clustering with individuals of Admixed American ancestry. Source data are provided as a Source Data file.

Figure S3

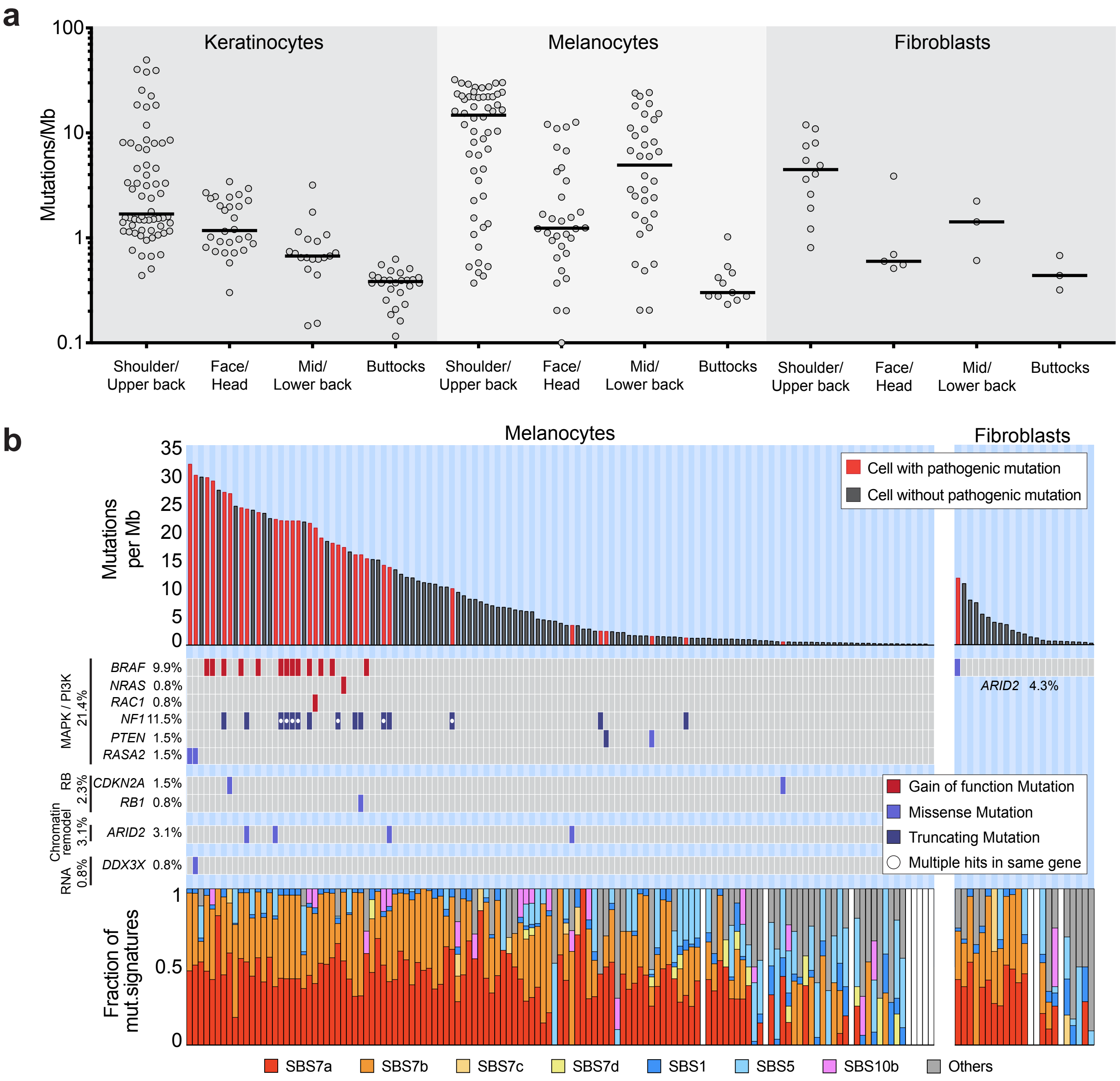

Figure S3. Melanocytes and fibroblasts have distinct mutational landscapes from keratinocytes. a Mutation burdens (mutations/megabase) of individual keratinocytes (left to right: n= 64, 28, 19, 26), melanocytes (left to right: n= 54, 32, 34, 11), or fibroblasts (left to right: n= 12, 5, 3, 3), stratified by their anatomic location. Black bars denote median values. **b** Top panel: mutation burden of melanocytes (left panel) and fibroblasts (right panel). Each column represents an individual cell, arranged in descending order of mutation burdens. Red bars indicate the presence of a pathogenic mutation(s) in that cell. Middle panel: tiling plot of pathogenic mutations (rows) in each cell (columns) with red tiles for gain of function mutation(s), light blue tiles for missense mutation(s), dark blue tiles for truncating (nonsense/splice-site/frameshift/indel) mutation(s) and a white circle for cells with multiple hits in the same gene. Bottom panel: the fractions of different mutational signatures attributed to each cell. White bars indicate cells with too few mutations to perform signature analysis. Source data are provided as a Source Data file.

Figure S4

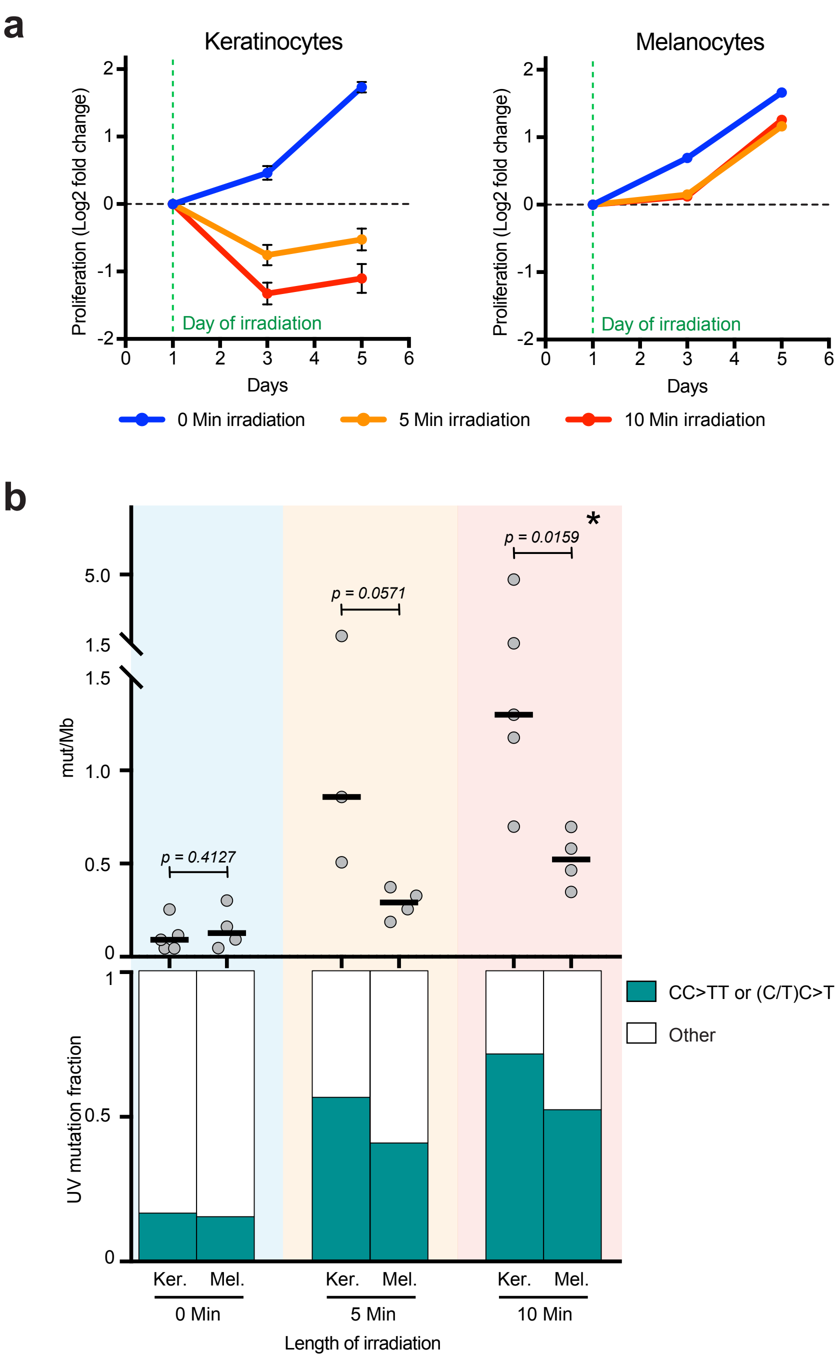

Figure S4. In vitro UV radiation of keratinocytes versus melanocytes. a The figure shows changes in cell count (log_2 fold change) over 5 days following UV irradiation for 0 (blue), 5 (orange), and 10 (red) minutes at 5.33 MED/hour in primary neonatal keratinocytes and melanocytes. Each data point represents mean +/- SD (n=3). **b** The top panel shows the mutation burden (mutations per megabase; mut/Mb) of individual keratinocytes (n=5,3,5) and melanocytes (n=4,4,4) following 0, 5, and 10 minutes of irradiation. Horizontal bars indicate the median. The bottom panel displays the proportion of UV-induced mutations—specifically CC>TT or (C>T)C>T—in each group. Cell type comparisons were assessed using the Wilcoxon rank-sum test. (*p<0.05). Source data are provided as a Source Data file.

Figure S5

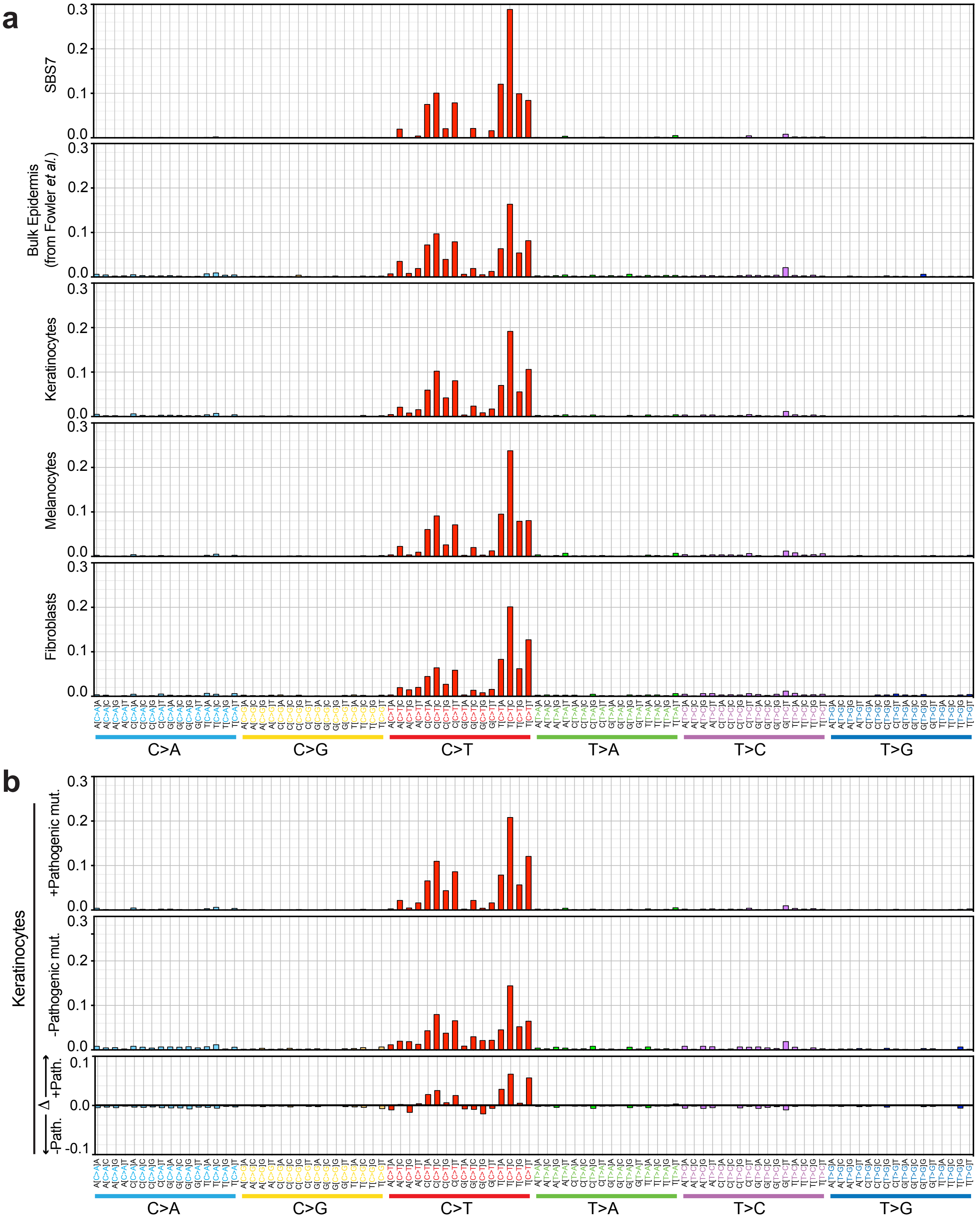

Figure S5. The tri-nucleotide context of single nucleotide point mutations in skin cells. 96-barplots show the frequency of the six potential somatic point mutations in all possible tri-nucleotide contexts. Panel **a** includes signature 7 as a reference as well as the cumulative frequencies of mutations, aggregated from normal skin biopsies (Fowler *et al.* 2021), and individual cells, of different skin cell types. Panel **b** shows the types of mutations in keratinocytes with and without underlying pathogenic mutations (Path.), aggregated from individual cells, and the differences between these groups. Source data are provided as a Source Data file.

Figure S6

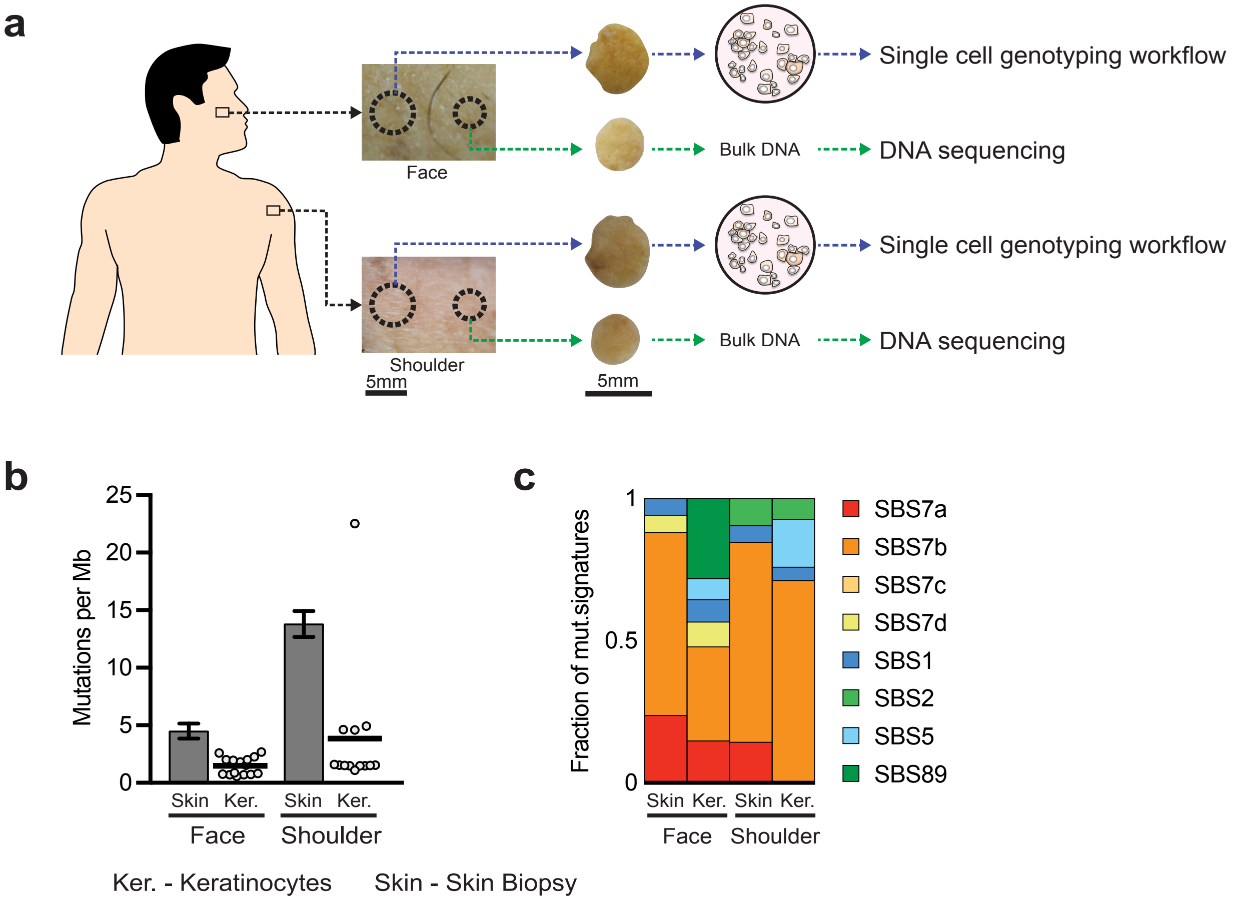

Figure S6. Mutational landscape of individual keratinocytes compared to adjoining skin biopsy. a The schematic illustrates the workflow for comparing the mutational profiles of keratinocytes derived from single-cell (Fig. S1a) and bulk sequencing approaches, using adjacent skin biopsies from the face and shoulder of the same donor (D56). **b** Mutation burdens (mutations per megabase) of individual keratinocytes from the face and shoulder are compared with those of adjacent skin biopsies. The mutation burden for skin biopsies is inferred from bulk sequencing data, as described by Martincorena *et al.* (Science, 2015). Error bars for skin biopsy mutation burdens represent 95% confidence intervals (Poisson test). For keratinocytes, each dot represents a single cell, and the horizontal bar indicates the mean mutation burden. **c** Stacked bar plots compare the mutation signatures of individual keratinocytes from face (n = 15) with the adjoining face skin biopsy and shoulder keratinocytes (n = 13) with the adjoining shoulder skin biopsy. Mutation signatures shown for individual keratinocytes are inferred from a compilation of all mutations in keratinocytes sequenced from the face and shoulder, respectively. Source data are provided as a Source Data file.

Figure S7

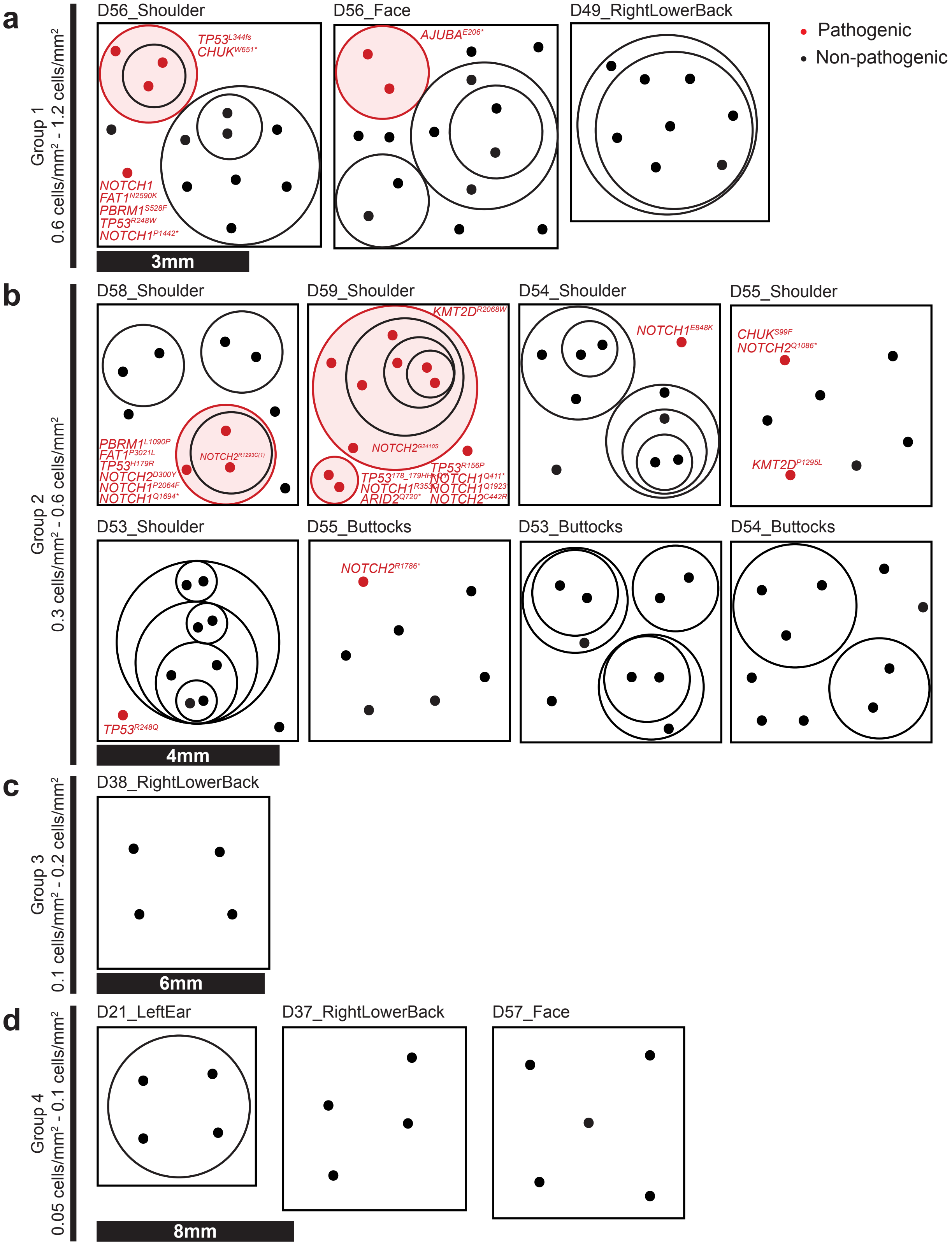

Figure S7. Clonal architecture of keratinocytes in human skin – the full sample set. a-d In figure 2a, we show the clonal structure of keratinocytes from a representative set of four biopsies. Here, we show all biopsies with more than 1 keratinocyte sampled at greater than 0.05 cells per square millimeter. Each schematic is plotted as described in figure 2a. Biopsies in each panel are grouped by their sampling densities, as indicated (most dense in panel a to least dense in panel d), with appropriate scale bars for each group. Source data are provided as a Source Data file.

Figure S8

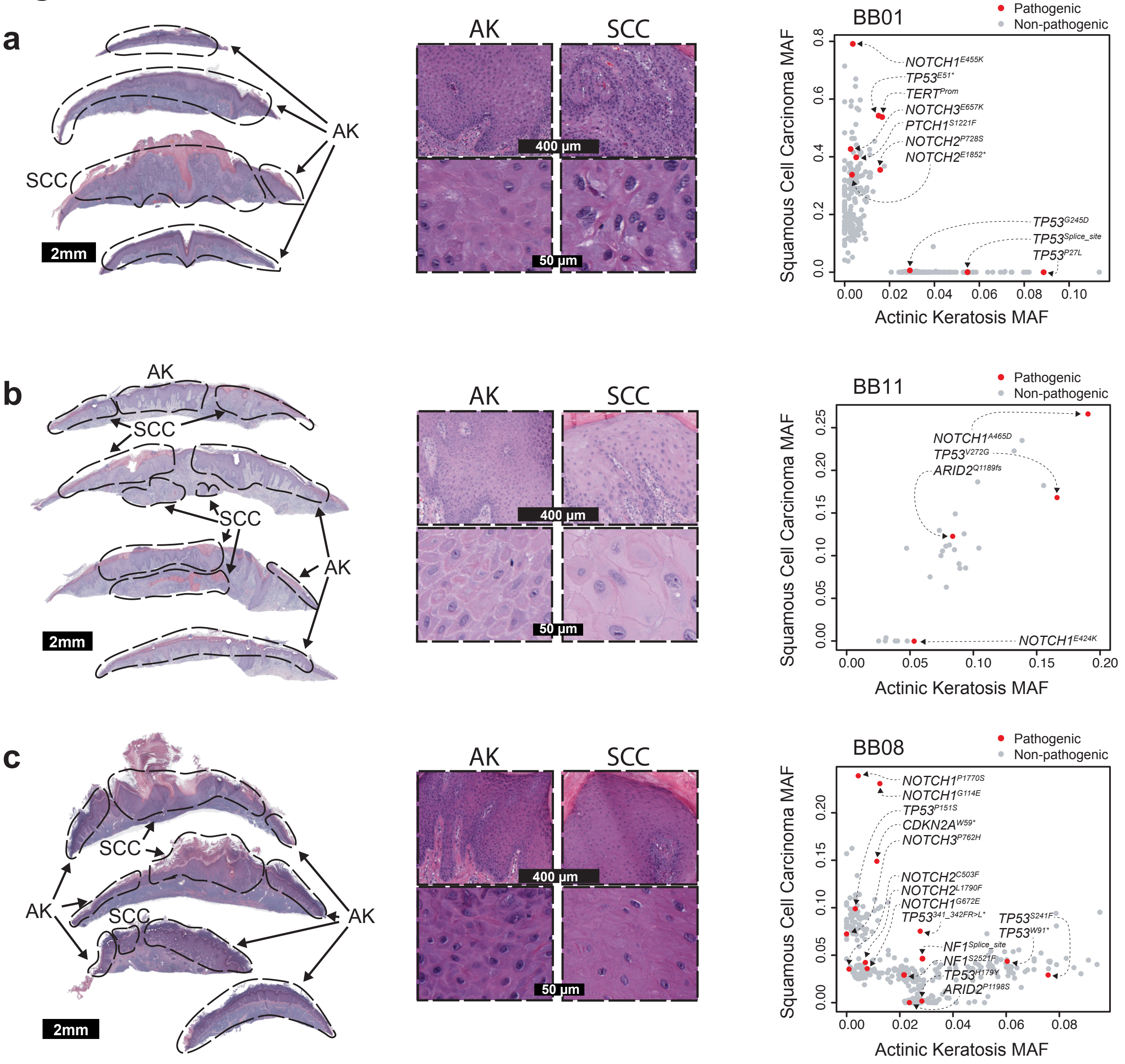

Figure S8. Examples of squamous cell carcinomas that did not evolve from the neighboring actinic keratoses. On the left-hand side of each panel, we show overview images and zoomed insets of H&E stained biopsies with histologically distinct areas. From each biopsy, we macrodissected areas with actinic keratosis (AK) histology or squamous cell carcinoma (SCC) histology, as indicated. On the righthand side of each panel, point mutations are stratified by their mutant allele fraction in each area with pathogenic mutations labeled. **a** A squamous cell carcinoma that does not share mutations with the neighboring actinic keratosis. This pattern suggests that the two neoplasms are not related. **b** A squamous cell carcinoma that shares mutations with adjacent tissue, but the squamous cell carcinoma lacks private mutations. This pattern implies that the adjacent tissue is a continuation of the squamous cell carcinoma, despite differences in their histologic appearance. **c** A complex case with multiple clones of keratinocytes whose phylogenetic relationship cannot be resolved. Source data are provided as a Source Data file.

Figure S9

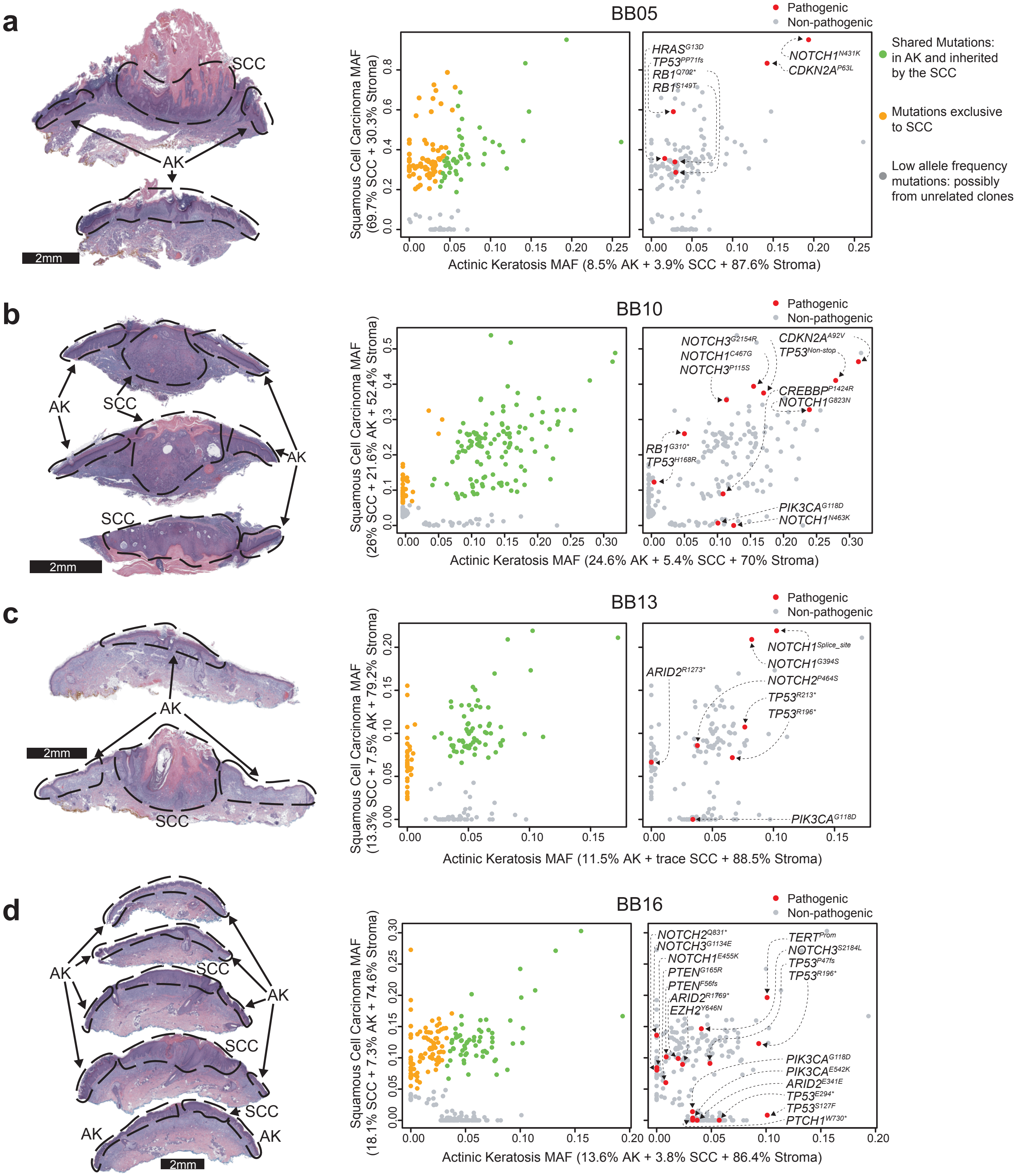

Figure S9. Examples of squamous cell carcinomas that evolved from neighboring actinic keratoses. The figure depicts cases: **a** BB05, **b** BB10, **c** BB13 and **d** BB16. For each case (**a-b**), the left image on the left presents overview images of H&E-stained biopsies with histologically distinct areas, macrodissected for squamous cell carcinomas (SCC) or actinic keratosis (AK), as indicated. The middle and right scatterplots display point mutations stratified by their mutant allele fractions (MAF) in each region. The middle plot shows mutations classified as shared (green) or exclusive to the SCC (orange) (see Methods for details). The right plot presents the same mutations, with pathogenic variants labeled. Source data are provided as a Source Data file.

Figure S10

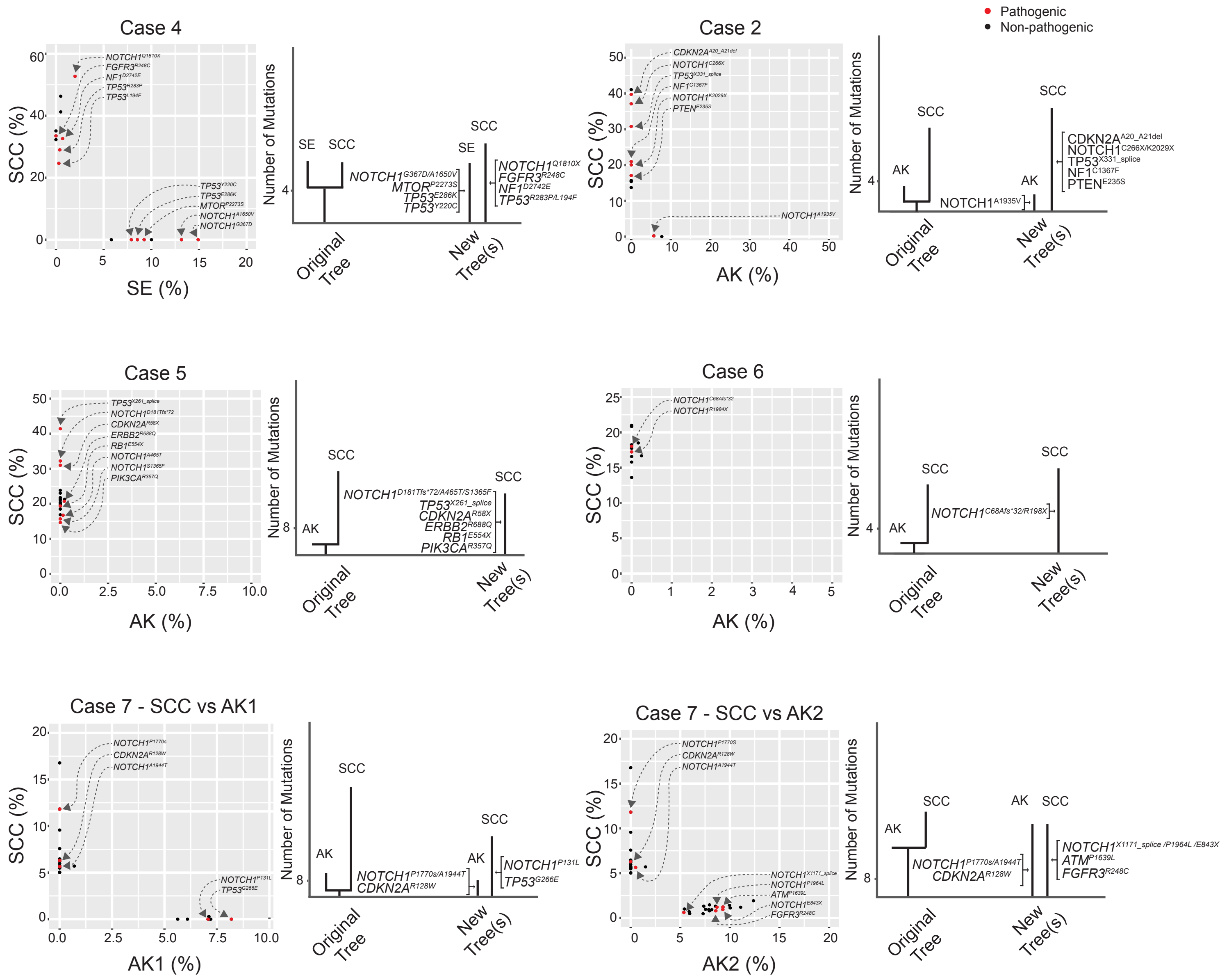

Figure S10. Examples of squamous cell carcinomas, from publicly available data, that do not share mutations with neighboring tissue. Kim *et al. JID*, 2022 sequenced squamous cell carcinomas (SCCs) and adjacent tissue. The adjacent tissue was classified as one of the following: actinic keratosis (AK), squamous cell carcinoma in situ (SCCIS), or sun-exposed skin (SE). Mutations were plotted by their allele frequencies in the adjacent tissue (x-axis) versus the squamous cell carcinoma (y-axis). Phylogenetic trees from the original study were reinterpreted by our group (see methods). In this series of cases, we do not believe that the dominant clone in the adjacent skin (x-axis) shares mutations with the dominant clone in the squamous cell carcinoma (y-axis). The mutations that were assigned to the trunks of trees in the original study tended to have high allele frequencies in one tissue but only trace sequencing reads in the other tissue, which likely stemmed from minor levels of contamination. Taken together, we conclude that the dominant clones in the squamous cell carcinomas were unrelated to the dominant clones in the neighboring tissue, and therefore, these clones arose independently, despite their proximity. Source data are provided as a Source Data file.

Figure S11

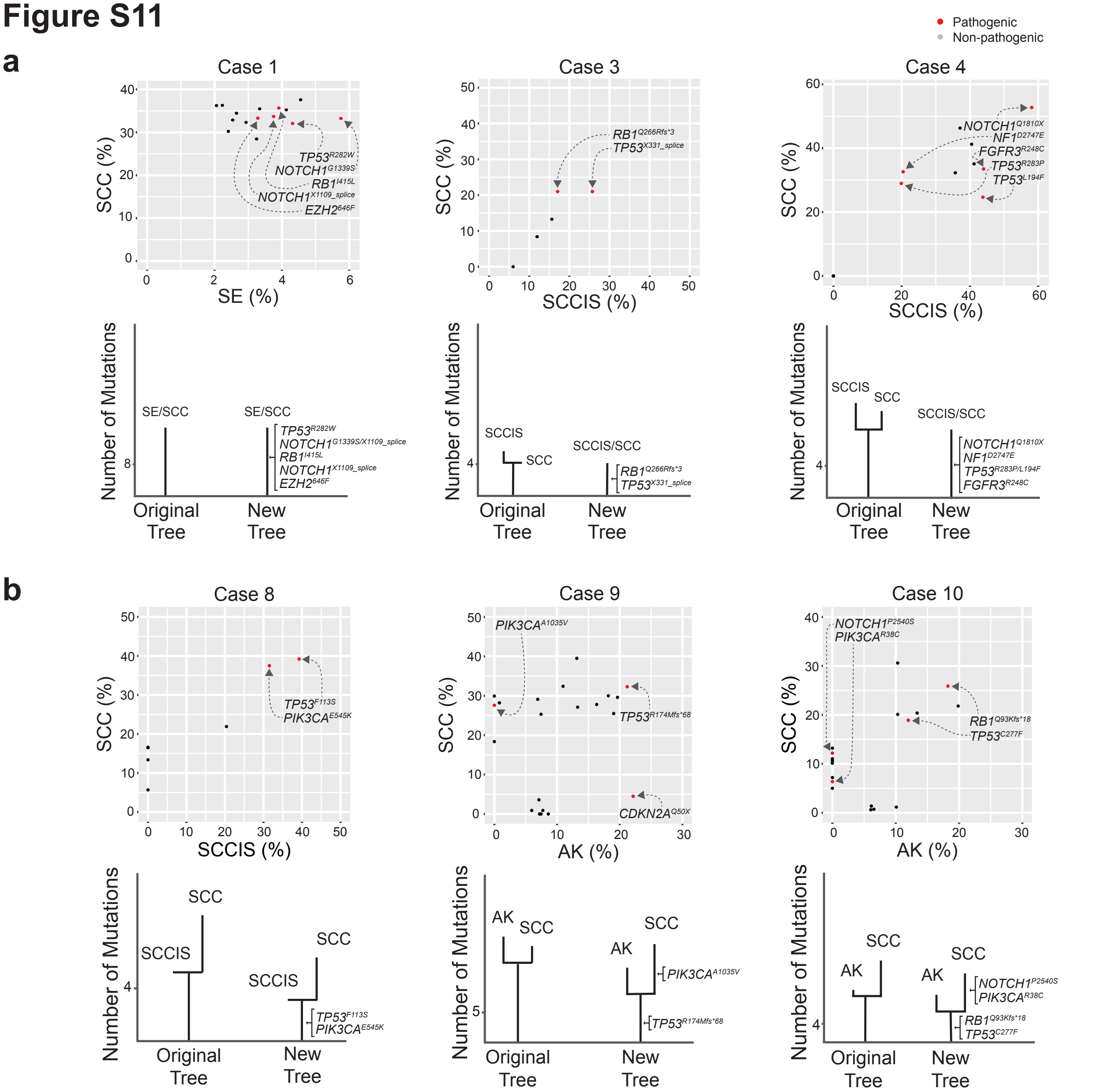

Figure S11. Additional examples of squamous cell carcinomas from publicly available data, some of which evolved from neighboring precursor lesions. Kim *et al. JID*, 2022 sequenced squamous cell carcinomas (SCCs) and adjacent tissue. The adjacent tissue was classified as one of the following: actinic keratosis (AK), squamous cell carcinoma in situ (SCCIS), or sun-exposed skin (SE). Mutations were plotted by their allele frequencies in the adjacent tissue (x-axis) versus the squamous cell carcinoma (y-axis). Phylogenetic trees from the original study were reinterpreted by our group (see methods). **a** Cases where the SCC are mutationally indistinguishable from neighboring tissue. In these cases, we did not find compelling evidence for branch mutations, private to either the precursor or descendent lesions. While there are mutations exclusively found in the SCCIS or SCC in cases 3 and 4, their allele frequencies were much lower than the mutations in the dominant clones in each tissue; therefore, we could not rule out the possibility that they came from an unrelated clone of cells, contaminating the macrodissected tissue. Taken together, we conclude the SCC to be mutationally indistinguishable from the dominant clone in the adjacent skin for these three cases. **b** Cases where the SCC evolved from the neighboring tissue. In cases 8, 9 and 10, there were shared mutations between the two tissues, suggesting that the squamous cell carcinomas were related to the neighboring tissues. There were also mutations exclusive to the squamous cell carcinomas, suggesting that they underwent an additional wave of clonal expansion. Taken together, we generally agree with the authors original interpretation for these cases that these squamous cell carcinomas evolved from neighboring precursor lesions. Source data are provided as a Source Data file.

Figure S12

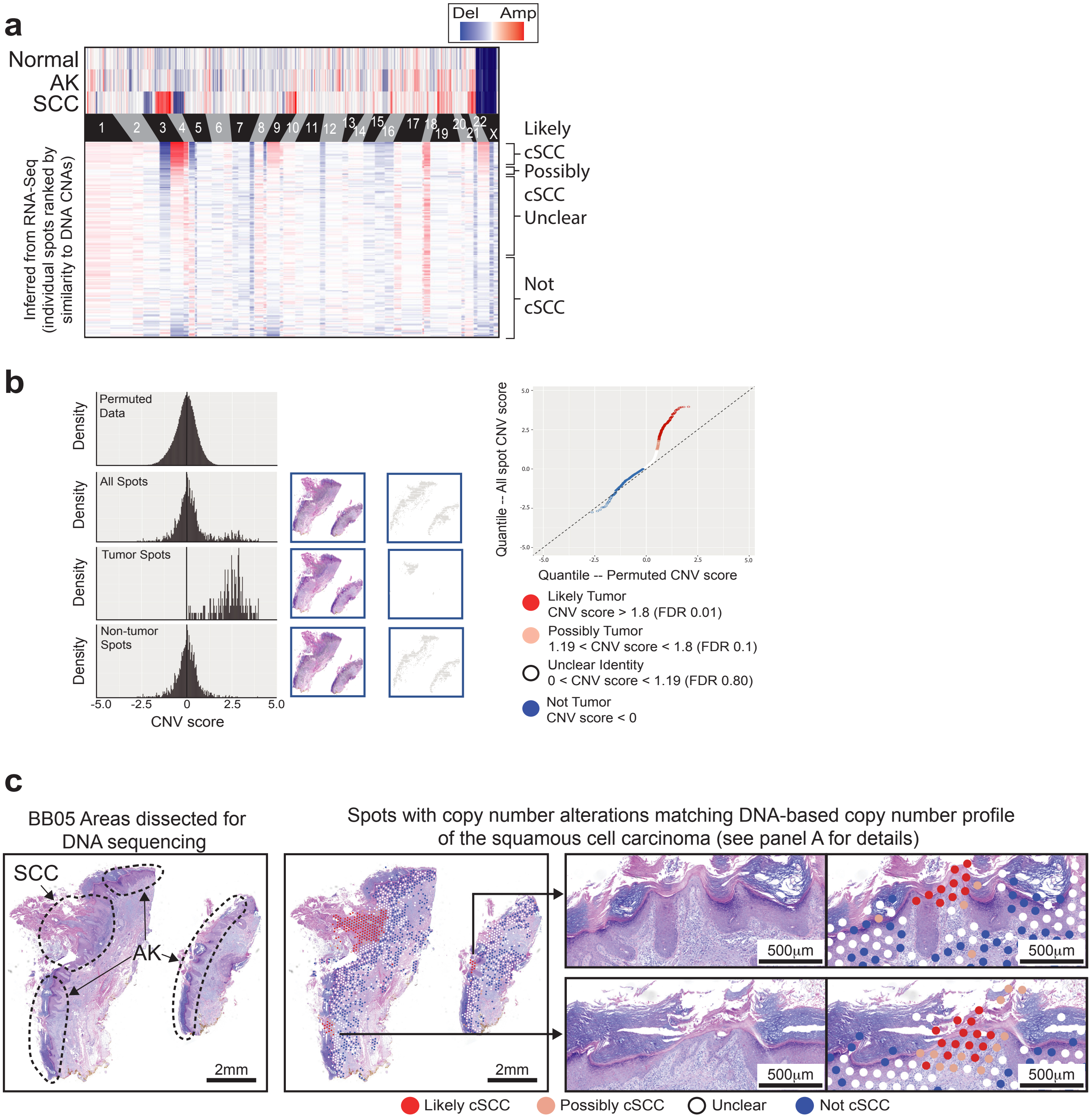

Figure S12. Copy number alterations are detectable in spatial transcriptomic data. a Copy number alterations (CNAs) were inferred from DNA-Sequencing data (top heatmap) and from RNA-sequencing data of individual spots (lower heatmap). Spots (rows in the lower heatmap) are ranked ordered by the similarity of their copy number profiles to the DNA copy number alterations. **b** For each spot in panel a, we calculated a CNVscore (see Chen *et al. Genome Biology*, 2023) to capture the similarity of the spot's copy number profile to the copy number inferred from DNA-seq data. Histogram of CNVscores from permuted and observed data are shown. A quantile-quantile plot was generated, comparing observed CNVscores to permuted scores, from which we established q-values to determine which spots were Likely, Possibly, or Not tumor, as indicated. **c** The spatial distribution of spots as defined in panel b.

Figure S13

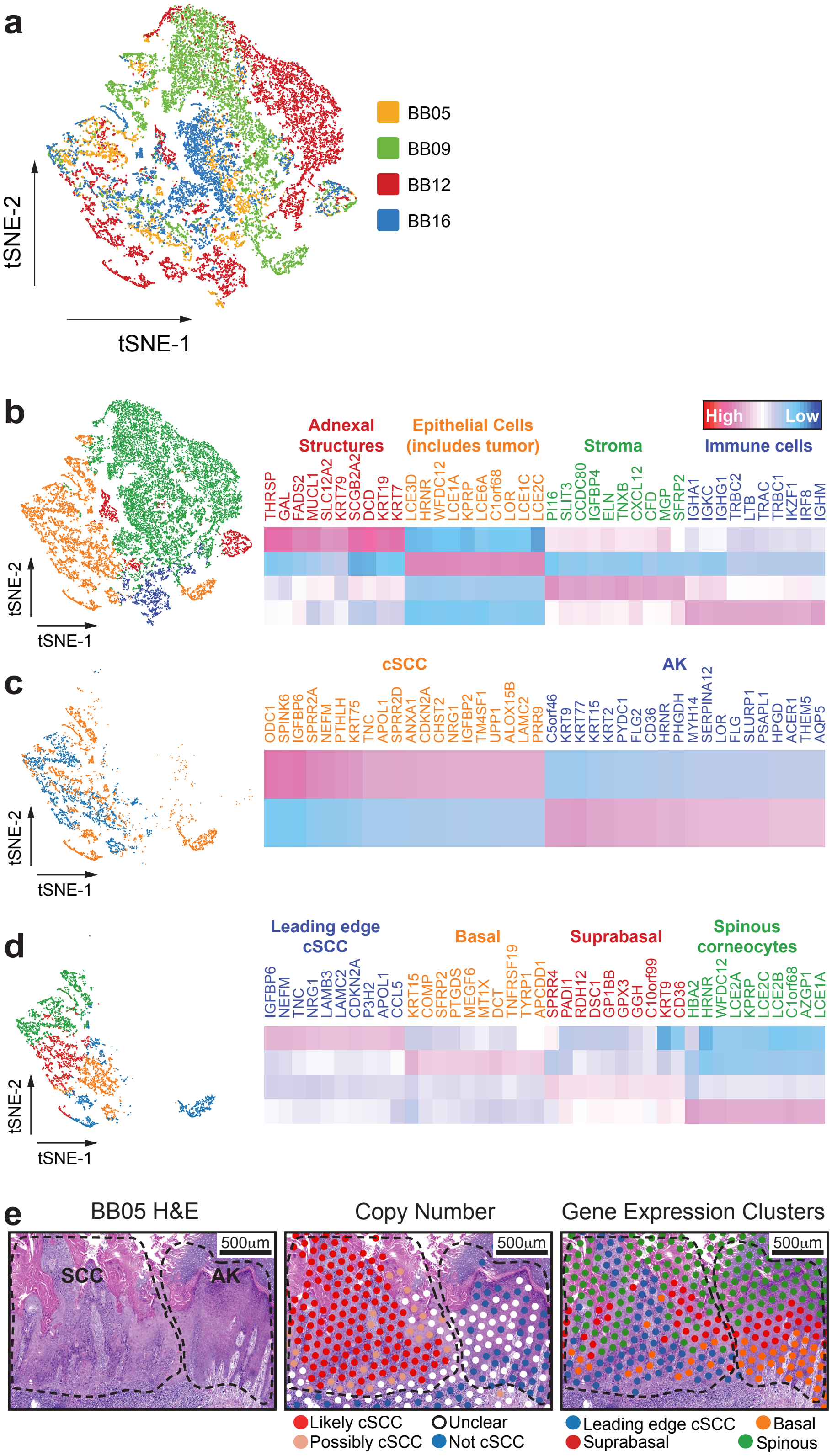

Figure S13. Spatial heterogeneity in gene expression during the evolution of squamous cell carcinoma from actinic keratosis. a A t-SNE plot of all spots from spatial transcriptomics data colored by sample ID. Spots clustered primarily by cell type or cell state, suggesting that batch effects were minimal. We performed spatial transcriptomics on a fifth case (BB13), but it was not included in this plot because it was profiled with an older version of Visium, whose design did not permit aggregate analyses with samples profiled on newer versions of the platform. **b-d** t-SNE plots of spots, aggregated from spatial transcriptomic data of four squamous cell carcinomas in association with actinic keratosis. Clusters of main cell types are annotated, as described, with the most highly expressed genes in each group highlighted in heatmaps to the right. **e** Zoomed view of the junction between squamous cell carcinoma (SCC) and actinic keratosis (AK) in BB05. H&E, copy number, and gene expression clusters are shown, as indicated. Note that keratinocytes in both the squamous cell carcinoma and actinic keratosis occupy a spectrum of differentiation states. Source data are provided as a Source Data file.

Figure S14

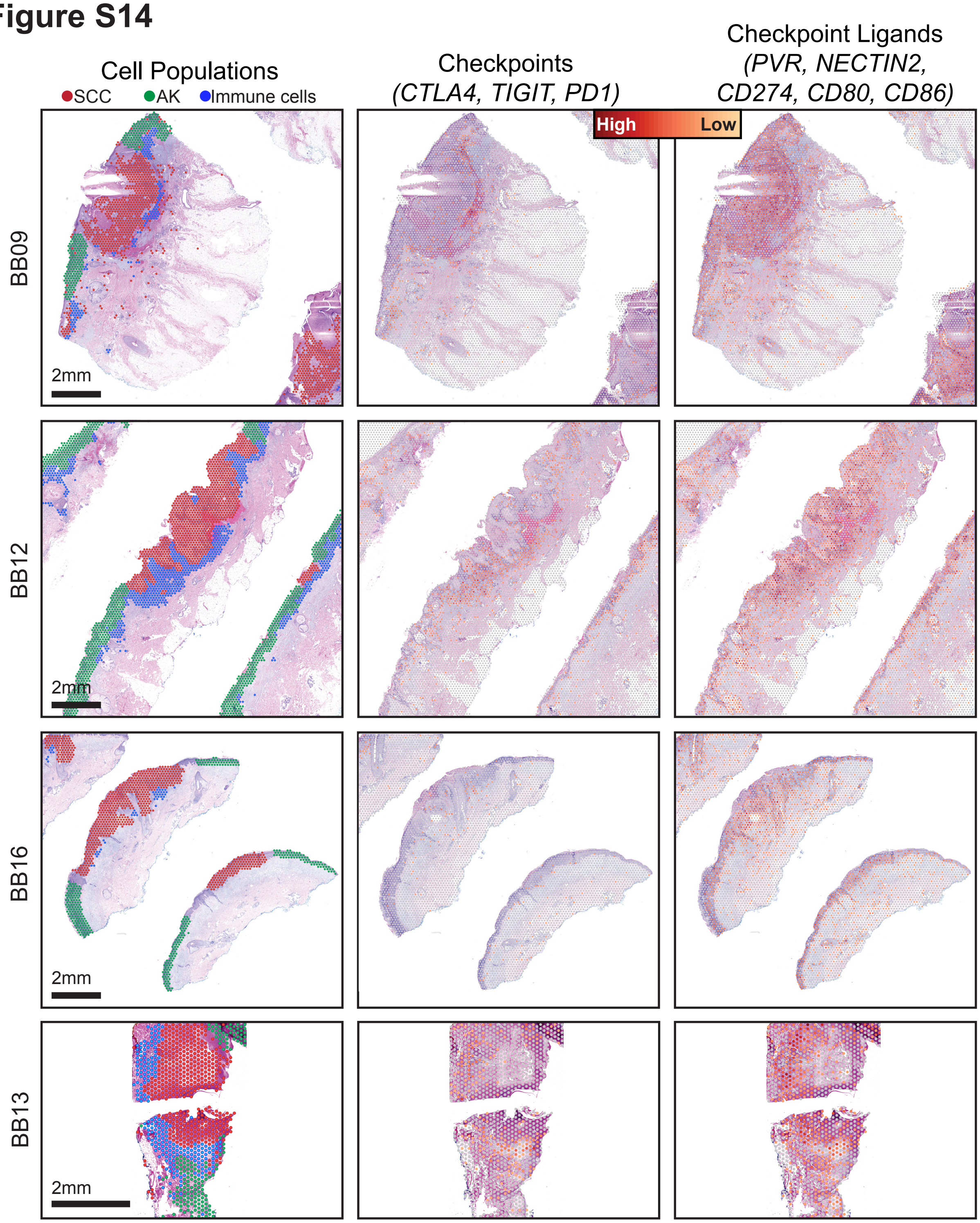

Figure S14. Spatial heterogeneity in gene expression of immune cells at the interface of squamous cell carcinoma (SCC) versus actinic keratosis (AK) – all cases. Each column of images shows a different view of spatial transcriptomic data, including: annotated spots and gene expression of immune checkpoints and their ligands. See figure 5 for an overview of the BB05. Gene expression intensities represent the combined expression of the checkpoint or ligand genes listed.
