# Supplementary material for: Genetic evolution of keratinocytes to cutaneous squamous cell carcinoma
Source: Nat Commun. 2025 Nov 27;16:10663. doi: 10.1038/s41467-025-65687-y (PMC12660313; doi:10.1038/s41467-025-65687-y)
Supplement: Supplementary file 3 — Description of Additional Supplementary Files [file 41467_2025_65687_MOESM3_ESM.pdf]

<u>Description of Additional Supplementary Files</u>

Supplementary Data S1. A summary of clinical, cytologic, genetic, and quality control metrics for individual skin cells sequenced in this study.

Supplementary Data S2. A summary of factors influencing sun exposure among study donors, including sex, age, ancestry, occupation, duration of time spent outdoors, exposure to occupational or environmental UV radiation, sunscreen use, biopsy site, and other relevant variables (if applicable).

Supplementary Data S3. List of point mutations in individual skin cells genotyped in this study.

Supplementary Data S4. A summary of clinical, histopathologic, genetic, and quality control metrics for each area of the cutaneous squamous cell carcinomas, in association with actinic keratoses, that were sequenced in this study.

Supplementary Data S5. Genes and bait intervals targeted for capture when sequencing cutaneous squamous cell carcinomas, in association with actinic keratoses.

Supplementary Data S6. List of point mutations detected in cutaneous squamous cell carcinomas, in association with actinic keratoses, that were genotyped in this study.
