# Supplementary material for: Genetic evolution of keratinocytes to cutaneous squamous cell carcinoma
Source: Nat Commun. 2025 Nov 27;16:10663. doi: 10.1038/s41467-025-65687-y (PMC12660313; doi:10.1038/s41467-025-65687-y)
Supplement: Supplementary file 10 — Reporting Summary [file 41467_2025_65687_MOESM10_ESM.pdf]

Corresponding author(s): A. Hunter Shain
Last updated by author(s): Sep 9, 2025

Reporting Summary

Nature Portfolio wishes to improve the reproducibility of the work that we publish. This form provides structure for consistency and transparency in reporting. For further information on Nature Portfolio policies, see our Editorial Policies and the Editorial Policy Checklist.

Statistics

For all statistical analyses, confirm that the following items are present in the figure legend, table legend, main text, or Methods section.

n/a	Confirmed	
☐	☒	The exact sample size (*n*) for each experimental group/condition, given as a discrete number and unit of measurement
☐	☒	A statement on whether measurements were taken from distinct samples or whether the same sample was measured repeatedly
☐	☒	The statistical test(s) used AND whether they are one- or two-sided<br>*Only common tests should be described solely by name; describe more complex techniques in the Methods section.*
☐	☒	A description of all covariates tested
☒	☐	A description of any assumptions or corrections, such as tests of normality and adjustment for multiple comparisons
☐	☒	A full description of the statistical parameters including central tendency (e.g. means) or other basic estimates (e.g. regression coefficient) AND variation (e.g. standard deviation) or associated estimates of uncertainty (e.g. confidence intervals)
☐	☒	For null hypothesis testing, the test statistic (e.g. *F*, *t*, *r*) with confidence intervals, effect sizes, degrees of freedom and *P* value noted<br>*Give P values as exact values whenever suitable.*
☒	☐	For Bayesian analysis, information on the choice of priors and Markov chain Monte Carlo settings
☒	☐	For hierarchical and complex designs, identification of the appropriate level for tests and full reporting of outcomes
☒	☐	Estimates of effect sizes (e.g. Cohen's *d*, Pearson's *r*), indicating how they were calculated

Our web collection on statistics for biologists contains articles on many of the points above.

Software and code

Policy information about availability of computer code

Data collection

No softwares or codes were used for data collection.

Data analysis

Code availability

All data analyses were conducted using publicly available software packages. The pipeline and tools for somatic mutation calling is available at [https://github.com/ShainLab/Single_Cell_Somatic_Mutation_Caller], a coverage analysis tool for counting the number of bases in the bam file with a specified coverage at [https://github.com/ShainLab/Footprints_v.0.1], script for identifying heterozygous Single Nucleotide Polymorphisms and haplotype phasing at [https://github.com/ShainLab/HaploPrep], the phylogenetic tree construction script at [https://github.com/ShainLab/Phylogenetic_tree], and the spatial transcriptomics package Stmut at [https://github.com/ShainLab/STmut]. All codes are publicly available under the MIT License.

For all the tools that are publicly available, detailed description of the softwares/tools used are as follows:

1. Sequencing and somatic alteration calls
Sequencing data from genomic DNA was aligned to the hg19 version of the genome with BWA (v2.0.5) and deduplicated with Picard (v2.1.1). Subsequently, the reads underwent additional curation to realign indels and recalibrate base quality using GATK (v4.1.2.0). For RNA sequencing data, alignment to both the genome and transcriptome was conducted with STAR align (v2.1.0). The reads were then deduplicated using Picard (v2.1.1) and gene-level read counts were quantified using RSEM (v1.2.0).
Copy number alterations were inferred from DNA- and RNA- sequencing data using CNVkit (v.0.9.6.2). A candidate set of germline heterozygous SNPs was called with FreeBayes (v.1.3.1) and further filtered to only include SNPs observed in the 1000 Genomes Project and between 40-60% allelic frequency mapping to each allele. A candidate set of short insertions and deletions was called with Pindel. A

candidate list of point mutations was called with MuTect2 (v4.1.2.0).

2. Mutation burden and mutational signature analyses
Mutation burdens were calculated as mutations per megabase. We counted the number of mutations as described above. To determine the footprint of genome with sufficient coverage in each clonal expansion, we calculated the footprints (as we described previously in Tang et al. Nature, 2020) to count the precise number of basepairs with 10X coverage or greater. For the analysis of mutational signatures, we compiled somatic mutations across all cells from both cohorts (Table S4) and established trinucleotide contexts for single base substitutions using the Bioconductor library BSgenome.Hsapiens.UCSC.hg19 (v1.4.3). This analysis was restricted to cells harboring a minimum of 10 mutations, as profiles with fewer mutations are statistically unreliable. . A custom forward stagewise algorithm using SigProfilerAssignment (v0.1.8) was applied to build a mutational profile based on 78 pre-defined COSMIC (v3.7) signatures previously extracted by SigProfiler 68. The minimum number of SBS mutations for the signature analysis is set at 10. The signatures for all the cells are depicted as stacked barplots in figure 1b (bottom panel) showing the fractions of top 7 signatures. Signatures present in less than 10% of cells were grouped into an [others] category.

3. Inference of cancer genome fraction in neoplastic tissues
Tumor genome fraction was inferred bioinformatically using same packages as above.

4. Spatial transcriptomics: Spatial transcriptomics was performed on five squamous cell carcinomas in association with actinic keratoses on a version of the 10X FFPE Visium platform. One case (BB13) was profiled on a relatively older version of Visium (v1.0). . The remaining cases (BB05, BB09, BB12 and BB16) were profiled with a relatively newer version of Visium (v2.0). Hybridization and preparation for sequencing was performed according to manufacturer's protocols by an outside company, Abiosciences. Paired-end sequencing was performed by the Center for Advanced Technology at UCSF on an Illumina instrument (NovaSeq 6000). Read 1 (the barcode read) was sequenced with a read length of 28 bp and read 2 (the probe read) was sequenced with a read length of 90 bp. Sequencing data was processed with the SpaceRanger pipeline (v1.3.0 and v2.0.1) to generate a cloupe file, which was visualized in the Loupe browser (version 7, 10X Genomics). The SpaceRanger workflow can run samples one-by-one or in aggregate mode. For the copy number analyses, we used STmut to infer copy number from individual spots. STmut can also accept lists of known copy number alterations and generate q-values for a given spot, which reflects the likelihood that it matches a known copy number profile. We called arm-level gains and losses from DNA-sequencing data and input these calls into STmut. For the gene expression analyses, we used the graph-based clusters generated by the SpaceRanger software.

For manuscripts utilizing custom algorithms or software that are central to the research but not yet described in published literature, software must be made available to editors and reviewers. We strongly encourage code deposition in a community repository (e.g. GitHub). See the Nature Portfolio guidelines for submitting code & software for further information.

Data

Policy information about availability of data

All manuscripts must include a data availability statement. This statement should provide the following information, where applicable:

- Accession codes, unique identifiers, or web links for publicly available datasets
- A description of any restrictions on data availability
- For clinical datasets or third party data, please ensure that the statement adheres to our policy

This study is part of the Human Tumor Atlas Network (HTAN), which is funded by the National Cancer Institute (U01 CA294536). The goal of HTAN is to catalog molecular transitions during the evolution of cancer. Raw and intermediate data are immediately available, as described below. These data will also be accessible through the HTAN data portal after the next data release (currently anticipated for Spring of 2025).

The DNA and RNA sequencing data of individual skin cells is available in dbGaP under accession codes phs001979.v1.p1 [https://www.ncbi.nlm.nih.gov/projects/gap/cgi-bin/study.cgi?study_id=phs001979.v1.p1] and phs003683.v2.p1 [https://www.ncbi.nlm.nih.gov/projects/gap/cgi-bin/study.cgi?study_id=phs003683.v2.p1]. The DNA sequencing data and spatial transcriptomic data from the cutaneous squamous cell carcinomas in association with actinic keratoses are available in dbGaP under accession code phs003282.v2.p1 [https://www.ncbi.nlm.nih.gov/projects/gap/cgi-bin/study.cgi?study_id=phs003282.v2.p1]. These accession numbers provide access to the raw sequencing FASTQ files.

Access to these datasets is restricted because participant consent permits data use only for biomedical research and does not allow unrestricted public release of individual-level genomic information. Investigators can request access through the dbGaP Data Access Committee via the dbGaP portal, and approved users receive data under institutional approvals and data use agreements consistent with the original consent. Requests are typically reviewed within 4–8 weeks, and data remain available for the duration of the repository's retention policy.

Intermediate levels of analysis are also available. Somatic mutation calls for individual cells are available in Supplementary Data S3 and were deposited in cBioPortal [https://www.cbioportal.org/study/clinicalData?id=normal_skin_keratinocytes_2024]. A summary of genetic alterations in each keratinocyte as well as copy number data from each cell is available on figshare: https://figshare.com/projects/Genetic_evolution_of_keratinocytes_to_cutaneous_squamous_cell_carcinoma/199837. Publicly available mutation data, covering the progression of squamous cell carcinoma from potential precursor lesions, was retrieved from Supplementary Table S3 of Kim et. al., JID, 2022. Publicly available mutation data, covering the somatic point mutations in epidermal biopsies was retrieved from Supplementary Table 2 (NIHMS63718-supplement-2.xlsx) of Martincorena et. al. Science, 2015. All analyses on publicly available data were performed with appropriate citation of the original source.

Source data are provided with this paper as the source data file.

Research involving human participants, their data, or biological material

Policy information about studies with human participants or human data. See also policy information about sex, gender (identity/presentation), and sexual orientation and race, ethnicity and racism.

Reporting on sex and gender	For this study, sex and gender was not part of the study design since our aim was to perform multi-omic profiling of epidermal keratinocytes from body sites that experience different degrees of habitual sun exposure. Skin was collected from the shoulders, buttocks, trunk, and head/neck area. Skin biopsies were collected from 15 unique donors (9 females and 6 males). The gender of living donors recruited for this study are self-reported. For cadavers, this information was based off medical records. All the details on the exact gender, age and other information on the donors are shared as a supplementary file (table S2).<br>However, samples from both male and female donors were included in all analyses to minimize sex-related biases in the results.
Reporting on race, ethnicity, or other socially relevant groupings	For this study, the only criterion was to analyze epidermal keratinocytes from various body sites. Therefore, race, ethnicity, age and other socially relevant groupings were not part of the study design. Donors ranged from 35-95 years of age and included Caucasians and Hispanics. For living donors recruited for this study; race, ethnicity and age are self-reported. For cadavers, this information was based off medical records.
Population characteristics	For the single-cell analysis of keratinocytes, samples were obtained from 15 donors ranging in age from 35 to 95 years. The cohort included 6 males and 9 females, with 14 individuals of European (White) ancestry and 1 individual of Hispanic/Latino ancestry. Additional donor details are provided in Table S2.<br><br>For the mutational profiling of squamous cell carcinomas associated with actinic keratoses, as well as spatial transcriptomics, samples were collected from 16 donors, including 11 males and 5 females, aged 57 to 94 years. Donor ethnicity was not disclosed. Additional details are provided in Table S4.
Recruitment	The samples for single-cell genomic analysis were collected from two sources:<br><br>1. Patients seen by Dr. Pedram Gerami at Northwestern University: During their clinic visits, patients were asked for consent to provide samples for this study. Only identifiers relevant to the study such as age, sex, and anatomical site were recorded. These patients were recruited from a high-risk skin cancer clinic and may therefore have a higher baseline risk for skin cancer than the general population. However, all samples collected were from normal skin, and site-matched comparisons between cell types were performed, minimizing any potential effect of this factor.<br><br>2. Donors from the UCSF Willed Body Program: These donors provided consent, prior to their death, for the use of their tissues for research or educational purposes. Donors in this cohort tend to be older than the general population. As with the first cohort, site-matched comparisons between cell types were performed, minimizing potential confounding effects.<br><br>Combining these two cohorts provides a more representative sample of the general population and helps mitigate cohort-specific biases, such as cancer risk and age.<br><br>Samples for mutational profiling of squamous cell carcinomas associated with actinic keratoses and spatial transcriptomics were obtained from the UCSF Dermatopathology Service archive. All patients had previously consented to the use of their samples for research purposes. Samples were selected solely based on the presence of squamous cell carcinoma adjacent to actinic keratoses. The selection process was unbiased with respect to other case characteristics, ensuring no introduction of additional confounding factors.
Ethics oversight	Living patients provided informed consent to participate in this study under protocols approved by the following institutional review boards: University of California, San Francisco institutional review board (IRB #22-36678) and Northwestern University institutional review board (IRB #STU00211546). Cadaver tissue came from donors who broadly consented, prior to their death as part of their living will, to the use of their tissues for medical research and/or educational purposes. Donors from the UCSF Willed Body Program were approved by, which facilitates the donation of individuals' remains for medical research. All donors had provided consent as part of their living will prior to their death.

Note that full information on the approval of the study protocol must also be provided in the manuscript.

Field-specific reporting

Please select the one below that is the best fit for your research. If you are not sure, read the appropriate sections before making your selection.

☒ Life sciences ☐ Behavioural & social sciences ☐ Ecological, evolutionary & environmental sciences

For a reference copy of the document with all sections, see nature.com/documents/nr-reporting-summary-flat.pdf

Life sciences study design

All studies must disclose on these points even when the disclosure is negative.

Sample size	In total, we measured somatic mutations from 137 keratinocytes, 131 melanocytes, and 23 fibroblasts from 22 different skin biopsies from 15 unique donors. The sample size for keratinocytes were based on all available genomic data. The sample size for melanocytes and fibroblasts

depended on site-matched comparisons to keratinocytes in this study as well as availability of genomic data.

Data exclusions	No data was excluded from the analyses.
Replication	In vitro experiments were not performed and therefore replication is not applicable. (see sample size description for a justification of the scope of the study).<br>To verify the reproducibility of our experimental findings, we implemented the following measures for each study subsection:<br><br>a. Genotyping keratinocytes, melanocytes, and fibroblasts<br><br>Donor level: Cells of each type were site-matched, with emphasis placed on minimizing confounding variables such as donor cancer history, anatomical site, sex, and age. Detailed donor information is provided in Table S2 for full transparency. Each cell analyzed corresponds to a complete sequencing run. In total, we profiled somatic mutations in single-cell expansions of 137 keratinocytes, 131 melanocytes, and 23 fibroblasts derived from 22 skin biopsies across 15 unique donors. Donors ranged in age from 35 to 95 years, and the cohort included 14 individuals of European ancestry and 1 of admixed American ancestry. This sample size provides a robust and reproducible genomic landscape for these cell types.<br><br>Cellular/genomic/transcriptomic level: We controlled for factors such as cell type, colony size, sequencing coverage, probes used, sensitivity, and specificity. These details are documented in Table S1 for added clarity.<br><br>Irradiation experiments: All cells used under each condition were from the same neonatal sample and passage number. Replicates were processed in parallel, under identical culture and sequencing conditions, to ensure reproducibility.<br><br>b. Mutation profiling of squamous cell carcinomas (SCCs) with associated actinic keratoses (AKs)<br>We collaborated with dermatopathologists to identify 20 SCC cases with adjacent AKs from the UCSF Dermatopathology Service archives. These included examples with complete overlap, partial overlap, and no overlap in somatic mutations. In cases where SCCs were associated with AKs, we reinforced our findings by integrating data from previously published studies (e.g., Kim et al.), further strengthening our conclusions.<br><br>c. Spatial transcriptomics<br>We repeated our spatial transcriptomic analysis across five independent cases, including one where SCCs and AKs were unrelated. The findings were consistent across replicates, confirming the reproducibility of the results.
Randomization	Randomization is not applicable to this study, as molecular comparisons focus on epidermal keratinocytes from body sites that experience different degrees of habitual sun exposure.
Blinding	Similar to randomization, blinding is not applicable to this study. The study's results are empirical, and the researchers have no influence on the outcome.

Reporting for specific materials, systems and methods

We require information from authors about some types of materials, experimental systems and methods used in many studies. Here, indicate whether each material, system or method listed is relevant to your study. If you are not sure if a list item applies to your research, read the appropriate section before selecting a response.

Materials & experimental systems

n/a	Involved in the study
☐	☒ Antibodies
☒	☐ Eukaryotic cell lines
☒	☐ Palaeontology and archaeology
☒	☐ Animals and other organisms
☒	☐ Clinical data
☒	☐ Dual use research of concern
☒	☐ Plants

Methods

n/a	Involved in the study
☒	☐ ChIP-seq
☒	☐ Flow cytometry
☒	☐ MRI-based neuroimaging

Antibodies

Antibodies used	1. TP53 (clone DO-7 mouse, Roche, 1:400 dilution)<br>2. Phospho-MAPK for Phospho-p44/42 MAPK (Erk1/2) (Thr202/Tyr204) (clone 4370, Cell Signaling, 1:100 dilution)
Validation	For TP53 antibody, cell signaling official website writes " This antibody has been validated using SimpleChIP® Enzymatic Chromatin IP Kits." The antibody is used by many prominent studies including the few listed below for IHC:<br><br>Carugo A, Minelli R, Sapio L, Soeung M, Carbone F, Robinson FS, Tepper J, Chen Z, Lovisa S, Svelto M, Amin S, Srinivasan S, Del Poggetto E, Loponte S, Puca F, Dey P, Malouf GG, Su X, Li L, Lopez-Terrada D, Rakheja D, Lazar AJ, Netto GJ, Rao P, Sgambato A, Maitra A, Tripathi DN, Walker CL, Karam JA, Heffernan TP, Viale A, Roberts CWM, Msaouel P, Tannir NM, Draetta GF, Genovese G. p53 Is a Master Regulator of Proteostasis in SMARCB1-Deficient Malignant Rhabdoid Tumors. Cancer Cell. 2019 Feb 11;35(2):204-220.e9. doi: 10.1016/j.ccell.2019.01.006. PMID: 30753823; PMCID: PMC7876656.

Pettinato AM, Yoo D, VanOudenhove J, Chen YS, Cohn R, Ladha FA, Yang X, Thakar K, Romano R, Legere N, Meredith E, Robson P, Regnier M, Cotney JL, Murry CE, Hinson JT. Sarcomere function activates a p53-dependent DNA damage response that promotes polyploidization and limits in vivo cell engraftment. Cell Rep. 2021 May 4;35(5):109088. doi: 10.1016/j.celrep.2021.109088. PMID: 33951429; PMCID: PMC8161465.

Additional studies that used this antibody is listed here:
[https://www.cellsignal.com/products/primary-antibodies/p53-do-7-mouse-mab/48818?srsltid=AfmBOoqqU0DE4yq0-rgaXQkZi40zqh3wWeM8DyjCL-Y1M7ZcAq4BopLA].

Phospho-p44/42 MAPK (Erk1/2) is an extremely well known antibody that has 9890 citations according to cell signaling webpage:
[https://www.cellsignal.com/products/primary-antibodies/phospho-p44-42-mapk-erk1-2-thr202-tyr204-d13-14-4e-xp-rabbit-mab/4370?srsltid=AfmBOoqWEKojIouoVLKDIrIRkZ3KC7RLiLYwWiDIrV_yEYR2BGIouFhG]
Some manuscripts that used this antibody are follows:

Babu S, Chen J, Baron CS, et al. Specific oncogene activation of the cell of origin in mucosal melanoma. Nat Commun. 2025;16(1):6750. Published 2025 Jul 22. doi:10.1038/s41467-025-61937-1.

Kuehl M, Okabayashi Y, Wong MN, Gernhold L, Gut G, Kaiser N, Schwerk M, Gräfe SK, Ma FY, Tanevski J, Schäfer PSL, Mezher S, Sarabia Del Castillo J, Goldbeck-Strieder T, Zolotareva O, Hartung M, Delgado Chaves FM, Klinkert L, Gnirck AC, Spehr M, Fleck D, Joodaki M, Parra V, Shaigan M, Diebold M, Prinz M, Kranz J, Kux JM, Braun F, Kretz O, Wu H, Grahammer F, Heins S, Zimmermann M, Haas F, Kylies D, Wanner N, Czogalla J, Dumoulin B, Zolotarev N, Lindenmeyer M, Karlson P, Nyengaard JR, Sebode M, Weidemann S, Wiech T, Groene HJ, Tomas NM, Meyer-Schwesinger C, Kuppe C, Kramann R, Karras A, Bruneval P, Tharaux PL, Pastene D, Yard B, Schaub JA, McCown PJ, Pyle L, Choi YJ, Yokoo T, Baumbach J, Sáez PJ, Costa I, Turner JE, Hodgin JB, Saez-Rodriguez J, Huber TB, Bjornstad P, Kretzler M, Lenoir O, Nikolic-Paterson DJ, Pelkmans L, Bonn S, Puelles VG. Pathology-oriented multiplexing enables integrative disease mapping. Nature. 2025 Aug;644(8076):516-526. doi: 10.1038/s41586-025-09225-2. Epub 2025 Jul 18. PMID: 40681898; PMCID: PMC12350167.

Plants

Seed stocks	n/a
Novel plant genotypes	n/a
Authentication	n/a
